# Involuntary musical imagery as a component of ordinary music cognition: A review of empirical evidence

**DOI:** 10.3758/s13423-020-01750-7

**Published:** 2020-06-24

**Authors:** Lassi A. Liikkanen, Kelly Jakubowski

**Affiliations:** 1 Department of Digital Humanities, University of Helsinki, Helsinki, Finland; 2 Department of Music, Durham University, Durham, UK

**Keywords:** Mental imagery, Involuntary musical imagery, Involuntary memory, Earworms

## Abstract

Involuntary musical imagery (INMI) refers to a conscious mental experience of music that occurs without deliberate efforts to initiate or sustain it. This experience often consists of the repetition of a short fragment of a melody, colloquially called an “earworm.” Here, we present the first comprehensive, qualitative review of published empirical research on INMI to date. We performed an extensive literature search and discovered, in total, 47 studies from 33 peer-reviewed articles that met the inclusion criteria for the review. In analyzing the content of these studies, we identified four major research themes, which concern the *phenomenology, dynamics, individual differences,* and *musical features* of INMI. The findings answer many questions of scientific interest—for instance, what is typical in terms of INMI frequency, duration, and content; which factors influence INMI onset; and whether demographic and personality factors can explain individual differences in susceptibility and responses to INMI. This review showcases INMI as a well-established phenomenon in light of a substantial body of empirical studies that have accumulated consistent results. Although the populations under study show an unfavorable bias towards Western, educated participants, the evidence depicts INMI as a universal psychological phenomenon, the possible function of which we do not yet fully understand. The concluding section introduces several suggestions for future research to expand on the topic.

**Electronic supplementary material:**

The online version of this article (10.3758/s13423-020-01750-7) contains supplementary material, which is available to authorized users.

Conscious thoughts are a lucrative but challenging topic for experimental psychology research. Phenomena such as inner thoughts leave little tangible, behavioral trace and have thus often eluded scientific investigation. Attempts to study such thoughts in vivo, as they unfold, have been particularly rare in comparison to retrospective reporting methods.

This review considers the topic of involuntary musical imagery (INMI) from a cognitive psychology perspective. INMI is usually described as the experience of imagined music protruding into daytime consciousness, without deliberate efforts to initiate or sustain it. Repetition is a common element of the experience, resulting in the experience colloquially known as an “earworm,” or having a song “stuck” in one’s head (see Williams, 2015, for a discussion). The term “imagery” ties INMI to a distinct tradition of research on mental imagery (Finke, 1989; Kosslyn, 1980), and distances it somewhat from the related area of involuntary memory research (Mace, 2007) by emphasizing the active construction of musical memories over recall. Mental imagery covers the full spectrum of all sensory experiences relived in our mind without the need for external (perceptual) stimulation. Although this term implies visual imagery, auditory and olfactory “images” are also common forms of mental imagery (Godøy & Jørgensen, 2001; Halpern & Zatorre, 1999; Zatorre, Halpern, Perry, Meyer, & Evans, 1996).

Musical imagery research began in the 1980s, when it was convincingly demonstrated that behavioral correlates consistent with the existence of a musical mental image could be found and manipulated (e.g., Halpern 1988a, 1988b, 1988c). Research on this topic quickly expanded into the domain of cognitive neuroscience (Halpern & Zatorre, 1999; Zatorre et al., 1996), producing evidence that brain activation during imagined and perceived music was notably similar. Consequently, musical imagery became a valid and popular research subject situated within the larger domain of auditory imagery research (Hubbard, 2010; Reisberg, 1992). In the past 15 years, the study of musical mental imagery has witnessed the exponential rise of yet another subdomain—INMI. Although initially investigated as a relatively niche area of music psychology research, it is now apparent that INMI can provide unique insights that advance our understanding of broader cognitive functions of attention, memory, and consciousness.

In this paper, we based our analyses on a hierarchy of musical imagery phenomena—visualized in Fig. 1 and following some ideas presented by Williams (2015)—although our proposed terminology deviates somewhat from Williams’s. The present review focuses specifically on findings from publications investigating nonintrusive, nonhallucinatory, involuntary musical imagery. The vast majority of studies in this domain have focused on the repetitive form of such INMI (which Williams, 2015, calls “earworms”), although several studies have not clearly differentiated between “earworms” and single (nonrepetitive) episodes of musical imagery called musical mind pops (see Elua, Laws, & Kvavilashvili, 2012). Thus, we acknowledge that some conclusions drawn here may not be exclusive to the repetitive variety of INMI, but we see this as a necessary step for future work.

**Fig. 1:**
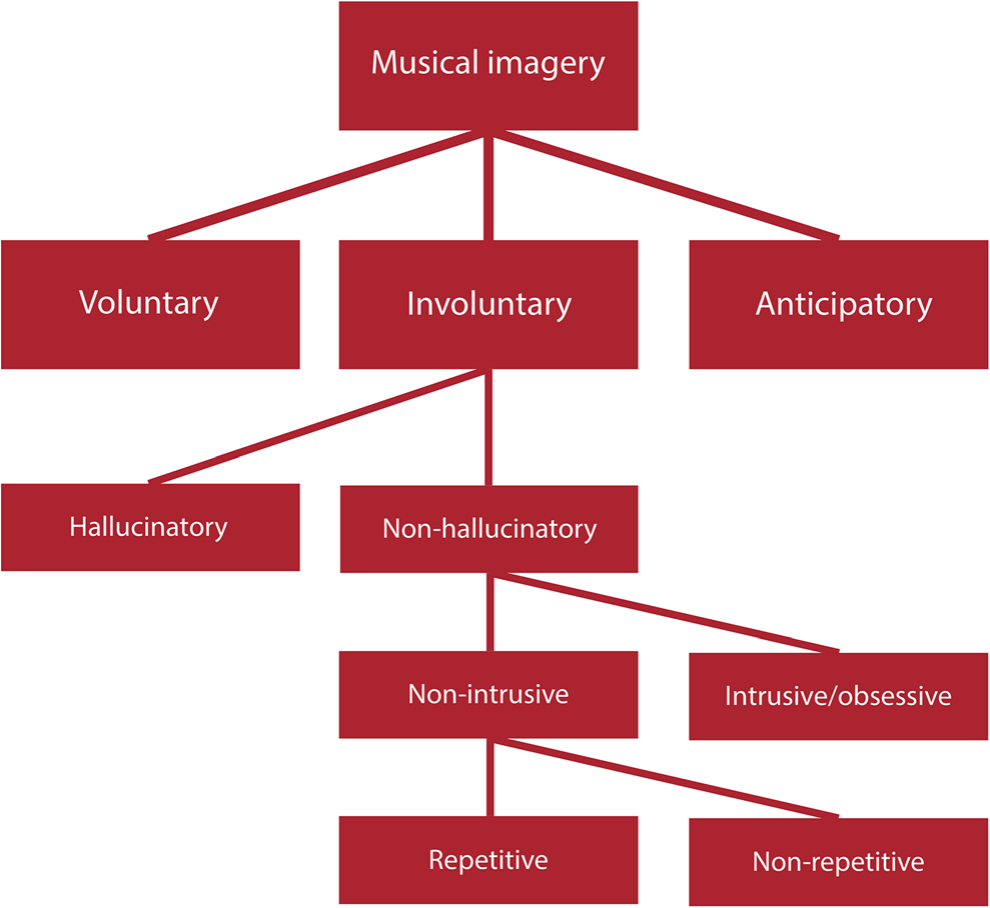
Hierarchy of musical imagery phenomena, adapted from Williams (2015)

## Motivation and objectives

Clinical uses and analysis of INMI formed an initial part of its short scientific history. Between the mid-1950s and early 2000s, a handful of papers within the psychodynamic tradition queried the meaning and interpretation of inner music (Freud, 1952; Kohut, 1957; Lipson, 2006; Reik, 1953; Saffe, 1983). This line of study has produced no cohesive body of research, but in the past 15 years or so, the topic has sparked the interest of cognitive psychologists. A body of empirical evidence on INMI has quickly accumulated and now warrants a review to highlight the key findings that numerous independent research groups have produced.

In this paper we investigate the INMI phenomenon through a literature review that covers the contemporary period of experimental psychology research on the topic from 2000 onwards. Our goal is to collect and analyze the contributions from all published psychological literature on INMI. The work is somewhat exploratory by nature, as the topic has not been similarly scrutinized in the past. Therefore, we aim to find a structure for thematically grouping and analyzing the diverse empirical findings as well as to review the methods that have been devised and adapted to study this phenomenon.

The remit of this review includes empirical studies that appear in peer-reviewed, psychology-oriented journals. We exclude studies in clinical psychology, in which we can find related topics that have long research traditions and can be considered related, but distinct phenomena. For instance, the psychiatric literature documents the phenomenon of *musical hallucinations* (American Psychiatric Association, 2013; Berrios, 1990; Evers & Ellger, 2004; Hermesh et al., 2004), which can be distinguished from *musical obsessions* (Taylor et al., 2014). Beyond these well-established disorders, there are phenomena such *pseudohallucinations* (van der Zwaard & Polak, 2001), *musical hallucinosis* (Griffiths, Jackson, Spillane, Friston, & Frackowiak, 1997), *musical palinacousis* (Di Dio, Fields, & Rowan, 2007), and *auditory Charles Bonnet syndrome* (Hori, Terao, & Nakamura, 2001), which all share some features with INMI (for a review, see Hemming & Merrill, 2015; Williams, 2015).

The aims of this review are to map out the aspects of INMI that have been systematically researched, to evaluate the degree to which research evidence converges across different studies and methodological approaches, and to assess the nature of INMI as a part of human cognitive function. We also suggest a structure for categorizing future studies and pinpoint key research questions that still require answers—something not warranted by looking at any individual empirical study in this domain in isolation.

## Method

### Study design and review protocol

The review was designed to be a comprehensive overview of all peer-reviewed, empirical psychological studies on INMI. We loosely followed the PRISMA protocol (Moher, Liberati, Tetzlaff, & Altman, 2009) to identify, screen, and select research papers. Our review is a qualitative one, as we explored the literature at large, rather than focusing on a single question that would be quantitatively weighted.

To identify potential papers for inclusion, two search strategies were used to gather a database of candidate publications. These were known, relevant literature citation analyses and online keyword searches. Known cornerstone articles from recent literature (e.g., Williams, 2015) were examined to identify relevant papers. In addition, a number of keywords (*earworm, earworms, involuntary musical imagery, tune in the head, stuck tunes, perpetual music track, musical mind pop*) were used to search selected publisher websites (Elsevier and Taylor & Francis), as well as Google Scholar and Web of Science, which also index other publishers such as APA and Wiley-Blackwell. In the keyword search, negative modifier keywords (*corn, agriculture*) were coupled when possible to remove irrelevant hits from the search results listings. In this review, we consider all papers published before May 1, 2019.

### Paper selection and data analysis

After collecting all INMI-related papers, we first screened them to select only studies that employed an empirical approach (including both quantitative and qualitative methods) situated within the psychological research tradition. This excluded, for instance, psychodynamic (Lipson, 2006), psychiatric (Liikkanen & Raaska, 2013), and humanities research (e.g., Priest, 2018) articles. In the next step, we considered the remaining papers eligible for inclusion only if they were peer-reviewed journal publications, thereby excluding a number of conference proceedings (such as Liikkanen 2008, 2009, and Lancashire, 2017), dissertations, and book chapters. This resulted in the exclusion of the very first attempts to utilize modern psychological methods to study INMI (Bennett, 2003; Kellaris, 2001, 2003), a book chapter describing an early induction experiment (Hemming, 2009), and three doctoral theses (Floridou, 2015; Liikkanen, 2018; McNally-Gagnon, 2016). Some studies that referred to INMI, but did not present new empirical results related to it, were also excluded (Bailes, 2006; Cotter & Silvia, 2019; Huovinen & Tuuri, 2019). One paper that we retained following the initial literature search (Bailes, 2007) made no explicit differentiation between INMI and voluntary musical imagery, but is discussed in the present review on the basis of its seminal contribution in establishing a methodology for capturing musical imagery experiences in everyday life. In addition, some papers identified in the initial search examined broader or related phenomena, such as involuntary memories and musical hallucinations (e.g., Beaty et al., 2013; Floridou et al., 2018; Moseley, Alderson-Day, Kumar, & Fernyhough, 2018), but were retained for the review on the basis that they reported novel results on INMI specifically.

The eligible papers were then closely analyzed, independently by the two authors. The studies within each paper were categorized based on the emerging research themes they touched upon, as well as according to the methods they employed. Individual studies could be categorized into more than one research theme and/or method if applicable. Only minor discrepancies in categorization occurred between the two authors, which were all successfully resolved via verbal discussion (at which point the final research theme names were also agreed upon). At this stage, we took into consideration the amount of convergent empirical evidence that was available to support each of the research themes. The final stage resulted in an exclusion of publications whose results fell outside of the review’s scope: being solely theoretical, or focusing on a theme of mental disorders for which we found inadequate support. The final set of papers that we consider in this review comprised 33 publications reporting 47 studies. A full list of excluded and included works that underwent the screening process after the initial search can be found in Supplementary Tables S1 and S2. Some of the excluded studies may be cited at appropriate occasions in the following text (e.g., Bailes, 2015), but they are not considered as the primary source of data for this review.

## Results and discussion

The 33 papers included in this review were categorized by their methods and research questions. It was common for a single publication to contain several studies or studies that involved multiple methods and/or research questions.

### Research methodology

Discovering methods that produce reliable data on INMI has been the key to progress in the field. Studies of INMI have met the methodological challenge of capturing a subjective, private mental experience with several solutions. The outcomes of these choices, rather than the full details of the methods, are assessed here because the methods themselves are not unique in empirical psychology research, although we provide below a short summary of how each of these methods have typically been applied in INMI research.

**Fig. 2:**
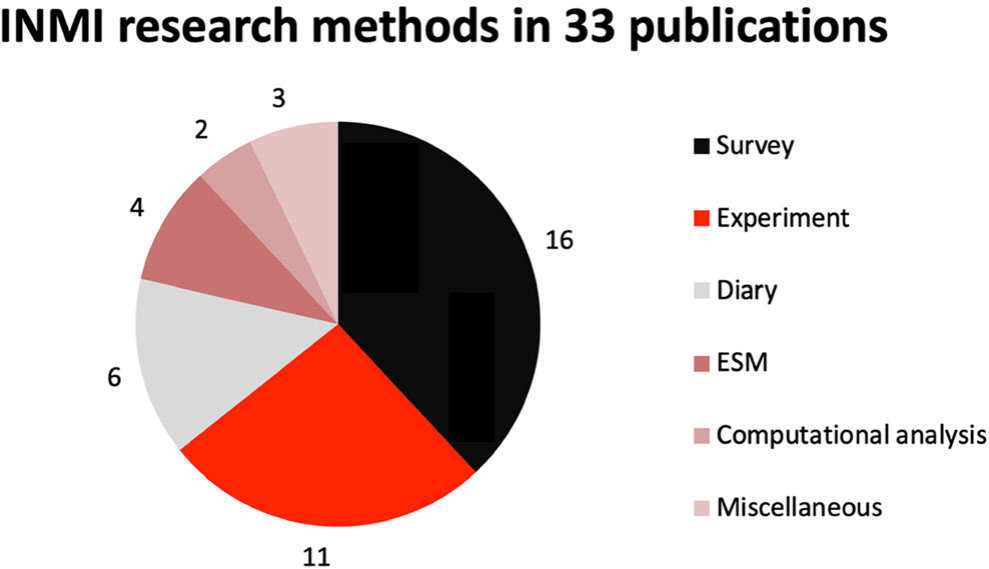
Research methods in the reviewed studies. (Note that some publications employed more than one method. ESM = experience sampling method)

The research methods employed in these studies are visualized in Fig. 2. They comprised five distinct approaches: surveys, experiments, diary studies, experience sampling method (ESM; Csikszentmihalyi & Larson, 1987) studies, and computational analyses. The bin “miscellaneous” included one of each of the following: interview study, case study, and a brain imaging study. Most studies utilized surveys or experiments, and completely novel survey designs prevailed over standardized instruments.

In INMI research, survey methods have most often been used to collect retrospective data on participants’ experiences of INMI, including such features as the duration, pleasantness, and distraction caused by INMI. These retrospective reports provide details about participants’ typical experiences, to the best of their memory (e.g., “On average, I get songs stuck in my head once per day”). Some standardized questionnaires to quantify the phenomenology of INMI have been developed. The Musical Imagery Questionnaire (MIQ; Baruss & Wammes, 2009) contains 42 individual items across six dimensions: *unconsciousness, persistence, entertainment, completeness, musicianship,* and *distraction*. However, the authors themselves concluded that the MIQ was not meaningful as a global measure of INMI. A more ambitious and further refined instrument called the Involuntary Musical Imagery Scale (IMIS) was created at Goldsmiths, University of London, by Floridou et al. (2015). In addition to questions on INMI frequency and duration, the IMIS contains four dimensions: *Negative valence, movement, personal reflections,* and *help [received]*. This instrument has been validated; the four subscales display high reliability (Cronbach’s alphas ranging from .76 to .91) and substantial retest correlations (from .65 to .79).

Experimental methods have most often been employed to induce INMI experiences. These usually involve efforts to prime INMI by presenting triggers to participants and measuring subsequent occurrences of INMI—for instance, first playing musical excerpts or presenting the lyrics to familiar songs followed by collecting reports of subsequent INMI that appear during an unrelated task (e.g., completing mazes or anagrams). Full details of this INMI induction procedure and its variants will be provided in the section titled Experimental Induction of INMI, below.

Another substantial body of research has probed the experience of INMI as it occurs in everyday life. This body of research can be further divided into studies using diary and ESM methods. In diary studies, participants are typically asked to record details about all INMI episodes experienced over a set time period (e.g., 1 week). During ESM studies, a researcher contacts each participant a set number of times (e.g., twice per day for 5 days) and asks them to report details of their current or recent INMI episodes. These naturalistic approaches have revealed insights on individual INMI episodes and the everyday situations surrounding their occurrence, including environmental triggers and concurrent activities. Finally, studies employing computational analysis have used such methods to analyze a large corpus of textual data describing INMI experiences (Liikkanen, Toivanen, & Jakubowski, 2015) and a corpus of pop songs typically reported as INMI (Jakubowski, Finkel, Stewart, & Müllensiefen, 2017).

#### Types and timing of self-report data collection methods

The most fundamental question in conducting INMI research is, “Can valid and reliable evidence on the subject be obtained?” All studies of INMI have relied on some variation of self-report methods, including both paper-based (e.g., Floridou & Müllensiefen, 2015) and digital (e.g., Liikkanen, 2012a) approaches. Most reporting has been direct and retrospective, which involves answering overt questions about musical imagery experiences with some delay, which has ranged from seconds (Beaty et al., 2013) to minutes (Bailes, 2007) in ESM studies, and may be substantially longer in survey studies. In rare instances, researchers have asked participants to document ongoing, concurrent INMI experiences—for example, by tapping to the beat of INMI and storing data on a wrist-worn accelerometer (Jakubowski, Farrugia, Halpern, Sankarpandi, & Stewart, 2015), or using an interactive voice response system (Beaty et al., 2013).

**Fig. 3:**
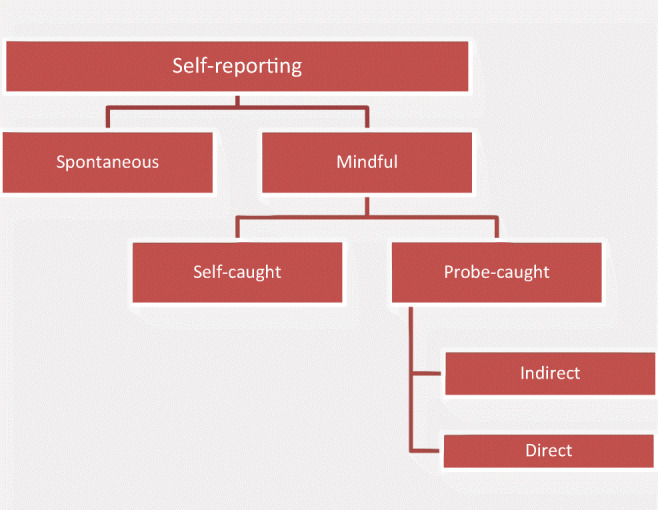
Approaches to self-report data collection on INMI experiences. Adapted from Liikkanen (2018)

Four methodological variations for collecting self-report data have been used in INMI research (see Fig. 3). Spontaneous reports are typically generated in nonexperimental settings and without direct questioning, but can be retrieved in the right kind of corpora (e.g., from social media; Liikkanen et al., 2015). Under research circumstances, people produce mindful reports that are either externally cued (probe-caught) or self-initiated (self-caught). Probed reports can further directly or indirectly address INMI, depending on whether the participants know the real aim of the study or not (Floridou, Williamson, & Stewart, 2017).

The concern as to the reliability of direct questioning about participants’ INMI experiences was raised by Floridou et al. (2017), who argued in favor of indirect questioning. Specifically, Floridou and colleagues asked participants to report details of a range of different thoughts and mental images, including INMI, that they had experienced during a 5-minute period. This was done to avoid directing participants’ attention to the fact that the study was particularly focused on INMI, and thereby minimize demand characteristics and other potential reporting biases. In addition, Baruss and Wammes (2009, p. 53) noted that by asking participants to describe INMI, the researchers may actually unintentionally induce INMI. This artifact is difficult to counter or quantify and remains a legitimate argument for the use of spontaneous or indirect measures; however, it is not supported by the results of Floridou et al. (2017), who found that indirect and direct questioning produced similar results in terms of the percentage of participants reporting INMI in their experiment.

Some studies (Beaman & Williams, 2010; Beaty et al., 2013) have compared retrospective reporting with ESM. Both studies concluded that retrospective reporting suffers from reporting biases. Beaty et al. (2013) wrote, “Retrospective studies can be useful for gathering preliminary data on a given phenomenon, but fall short of the ecological validity of sampling momentary experiences” (p. 1171). For instance, Beaty et al. (2013) found that participants in their retrospective survey reported experiencing musical imagery for “more than half of their waking hours” (p. 1165), whereas only 17% of the 4,403 completed experience sampling forms contained musical imagery reports.^1^ This particular study had a confound of automatically discarding imagery reported during music listening that may lead to mild underreporting, as Bailes (2007) found that 3% of participant reports in an ESM study involved simultaneous listening to and imagining of music. However, the results of Beaty et al. are similar to a study involving far fewer individuals (Beaman & Williams, 2010).

Finally, it seems that some phenomenological aspects of INMI are particularly sensitive to methodological choice. Halpern and Bartlett (2011) described major discrepancies between their participants’ estimations of INMI duration across two diary studies. They observed how different reporting requirements across studies resulted in as much as a four-fold increase in reported duration. Specifically, longer INMI episodes were reported in a diary study requiring more detailed reports of the experience in comparison with a second study using a shorter report form, indicating a possible tendency to prioritize reporting longer INMI episodes over more fleeting experiences in the first study.

#### Research methodology: Discussion

Methodological progress in INMI research has largely been achieved by adapting existing methods that have parallels in the involuntary thought and memory research traditions (see Hyman et al., 2015). These methods have proven that self-reporting can produce systematic, retest reliable results regarding INMI (Cotter, Christensen, & Silvia, 2016; Floridou et al., 2015). This was elegantly illustrated in a study that showed how results based on self-reports can be used to identify individual differences in cortical structure (Farrugia, Jakubowski, Cusack, & Stewart, 2015). Empirical studies have also produced novel methods for inducing and capturing INMI. For instance, recording INMI by tapping to its beat (Jakubowski, Bashir, Farrugia, & Stewart, 2018; Jakubowski et al., 2015) has provided strong evidence on the similarities between INMI, voluntary imagery, and perception.

The biggest remaining methodological gap concerns the sole reliance on self-report data. Unfortunately, the direct detection of the INMI experience—for instance, from brain or motor system activation—has not yet been demonstrated as feasible, even though certain aspects of musical imagery have been reliably observed in brain imaging (Kraemer, Macrae, Green, & Kelley, 2005). Given the findings of voluntary musical imagery research (Halpern & Zatorre, 1999; Halpern, Zatorre, Bouffard, & Johnson, 2004), it is predicted that INMI may reveal similar activations in auditory and frontal cortices to voluntary musical imagery (see Hemming & Merrill, 2015), as well as patterns previously witnessed in involuntary memory studies (Hall, Gjedde, & Kupers, 2008). In addition, recent work has shown that activity in laryngeal muscles recorded using surface electromyography may be an objective indicator of voluntary musical imagery (Pruitt, Halpern, & Pfordresher, 2019), suggesting that such methods may be promising for INMI research as well.

An additional point of methodological concern is the nonreplication of certain findings within the literature. For instance, one study (Beaman & Williams, 2013) found that several detailed results deviated from their prior work—for instance, a failure to identify any individual differences factors to explain the frequency of INMI reports (Beaman & Williams, 2010). Such anomalies may result from the fact that few studies have used standardized measures to quantify INMI and its phenomenology. It appears that small changes in a survey’s wording may quickly render single-item responses incomparable across studies. This is made more complicated by the fact that not all studies have fully disclosed the instructions, survey instruments, and experience sampling forms they have used to manipulate and measure INMI. Fortunately, a standardized survey instrument for INMI has recently been proposed (Floridou et al., 2015) and already adopted by other researchers (see Cotter et al., 2016; Farrugia et al., 2015), which may help to address this source of methodological variation. This survey utilizes scales rather than individual items, which further reduces variation in the measured dimension. It is also important that self-report instruments do not presume an either–or relationship between even closely related activities of music listening and imaging, as both may occur simultaneously (Bailes, 2007).

### Emerging research themes

Beyond summarizing the methodological approaches, a primary aim of this review was to categorize the research questions interrogated in INMI research. These were grouped under four themes, as follows:

1. Phenomenology of INMI
2. Dynamics of INMI
3. Individual differences in INMI
4. Musical features of INMI

The studies considering phenomenology examined questions on INMI’s existence: What are the experiences like in quantitative and qualitative terms? The dynamics of INMI theme refers to research that investigates how INMI experiences emerge, are maintained, or terminate; such investigations are typically made possible through experimental manipulations. A further step is taken in studies of individual differences, which attempt to reveal systematic differences in the phenomenology or dynamics of INMI between people. The fourth included theme is a distinct question on the importance of the features of the music in evoking INMI.

**Fig. 4:**
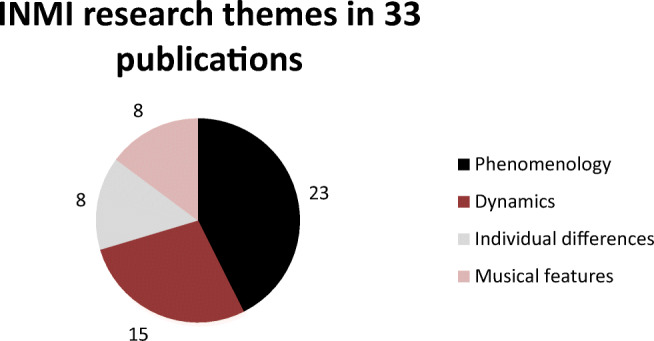
Research themes in the reviewed studies

A quantitative assessment of the research themes shows that *phenomenology* is by far the most studied theme, followed by *dynamics* (see Fig. 4). The remaining two themes were covered in around one quarter of the studies. The following sections synthesize the findings under each theme.

### Phenomenology of INMI

Studies of INMI phenomenology describe the characteristics and subjective experience of INMI. These descriptions have been predominantly quantitative and aggregated, with only a few exceptions describing individual experiences (Brown, 2006; Hemming & Merrill, 2015; Williamson & Jilka, 2014).

Four measures have been widely adopted to describe INMI: frequency, duration, veridicality, and subjective pleasantness. Frequency is the measure that appears in most publications. The simplest frequency measure is a binary indication of whether an INMI episode has been experienced or not.

#### Global recognition and frequency of INMI

Several studies indicate that people across the English-speaking world recognize INMI and spontaneously and publicly discuss their INMI experiences (Liikkanen et al., 2015; Williamson et al., 2012). Similar evidence can be found in French, German, and Finnish language-speaking countries (Hemming, 2008; Liikkanen, 2012c; McNally-Gagnon, 2016).

In a study of Twitter users’ INMI experiences, Liikkanen et al. (2015) collated 80,620 short social media messages known as tweets (see http://twitter.com/about) using a keyword search to isolate possible INMI-related content. Twenty-eight percent of the data were classified as reports of ongoing or past INMI experiences. These tweets came from 173 countries, indicating that English-speaking people across the world spontaneously report INMI experiences.

**Fig. 5:**
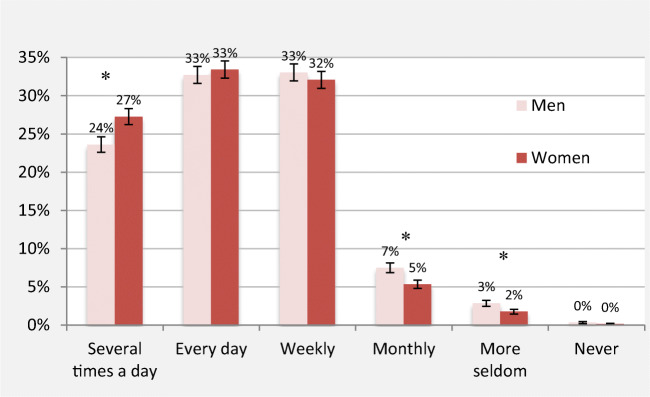
Self-reported frequency of INMI among women (*N* = 8,144) and men (*N* = 3,766). Error bars show 99% confidence intervals. An asterisk (*) denotes statistically significant differences between sexes (*p* < .01). Adapted from Liikkanen (2012c)

The most comprehensive accounts of INMI frequency are based on retrospective reporting. Liikkanen (2012c) reported that INMI was experienced at least every week by 92% of the Finnish respondents in a large, Internet-based sample (*N* = 11,910), as shown in Fig. 5. No large-scale studies have since tried to replicate this finding (see, e.g., Floridou et al., 2015; Müllensiefen et al., 2014, for smaller scale replications).

ESM studies also support the idea of frequent INMI experiences. The prevalence of episodes occurring at the time of probe ranges from 17% in a study of general musical imagery experiences (Beaty et al., 2013) to 47% in a study of INMI in particular (Floridou & Müllensiefen, 2015). This gives further credibility to the claims about the commonality of the phenomenon, at least among Western people.

#### Duration of INMI episodes

Estimating INMI duration via retrospective self-report methods can be problematic, and thus confident reports on this topic are few. In a novel instance of INMI duration measurement in an experimental setting (Moeck, Hyman, & Takarangi, 2018), participants were asked to hold down the computer keyboard space bar throughout the duration of any INMI episodes that they experienced, releasing it at the end of each episode. Individual INMI episodes lasted less than 10 seconds on average, and multiple episodes accumulated during a 5-minute thought-monitoring period lasted a mean duration of 44 seconds altogether.

Diary studies have reported INMI episodes lasting from under a minute to a median or average duration of 8, 27, or 36 minutes (Beaman & Williams, 2010; Halpern & Bartlett, 2011). Retrospective studies provide even longer duration estimates, from hours to days (Beaman & Williams, 2010). For instance, Hyman et al. (2015) have reported “a few hours” as the modal response for INMI duration. This indicates that experimental, diary, and survey methods result in different estimates of INMI duration.

#### Veridicality of recall in INMI

INMI has several features in common with voluntary musical imagery. One of these shared features is the high faithfulness of musical images. This has been a repeated finding in both in-depth qualitative studies (Brown, 2006; Williamson & Jilka, 2014) and quantitative research (Jakubowski et al., 2018; Jakubowski et al., 2015). Williamson and Jilka (2014) state that musical imagery is frequently “comparable to an actual music listening experience” (p. 667), and INMI experiences can be described as simplified versions of the canonical recording*.* In a study of the general musical imagery experiences of music students, Bailes (2007) reported higher vividness ratings for melody and lyrics over other musical features. She mentions the limitations of the human voice in reproducing timbre or texture, in comparison with pitch or words, as a possible explanation for this difference in the representation of musical features.

**Fig. 6:**
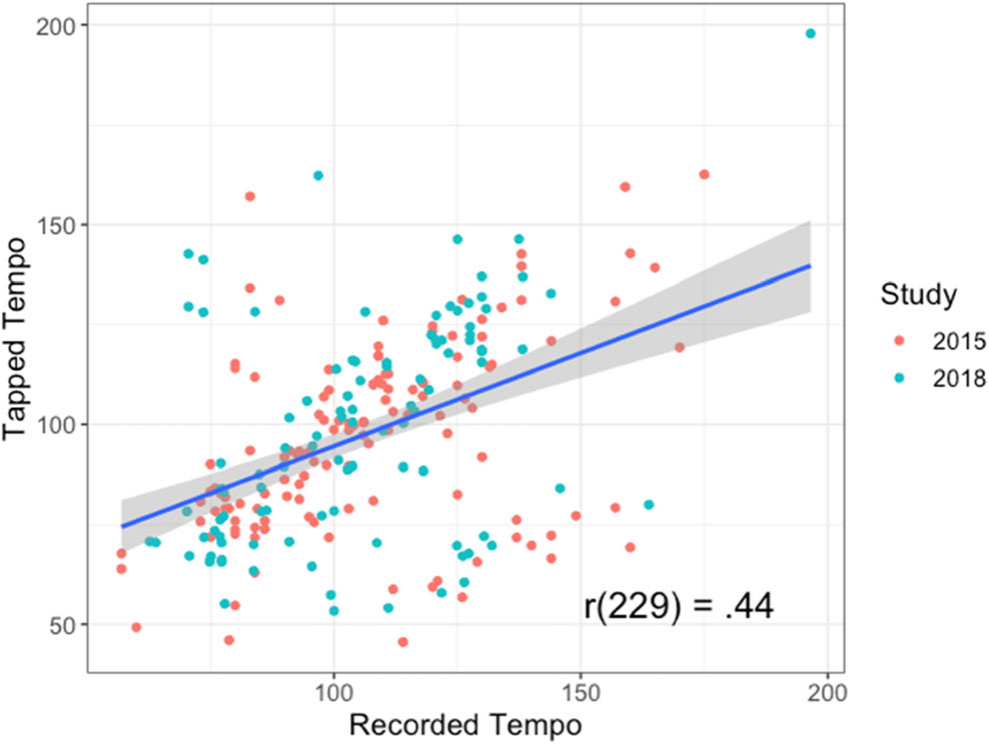
Scatterplot of the relationship between recorded (canonical) and tapped (imagined) tempo from two studies of Jakubowski et al. (2015, *N* = 130; Jakubowski et al. (2018), *N* = 101 episodes), adapted from the original figure

Going beyond self-report measures, the veridicality of INMI has also been behaviorally verified. The tempo of INMI songs has been investigated using an innovative technique of recording self-caught INMI episodes (Jakubowski et al., 2018; Jakubowski et al., 2015). In both studies, participants wore wristband accelerometers. They were instructed to use the associated hand to tap to the beat when they experienced INMI and record the details of the INMI incident in a diary. The tempi of INMI episodes were calculated from the tapping data, and it was found that INMI tempo deviated only 15% on average from canonical, recorded versions of the music (Jakubowski et al., 2015; see Fig. 6). These results were replicated in a subsequent study, which revealed that INMI did not significantly differ from voluntary musical imagery for the same songs in terms of veridicality of tempo recall (Jakubowski et al., 2018).

**Fig. 7:**
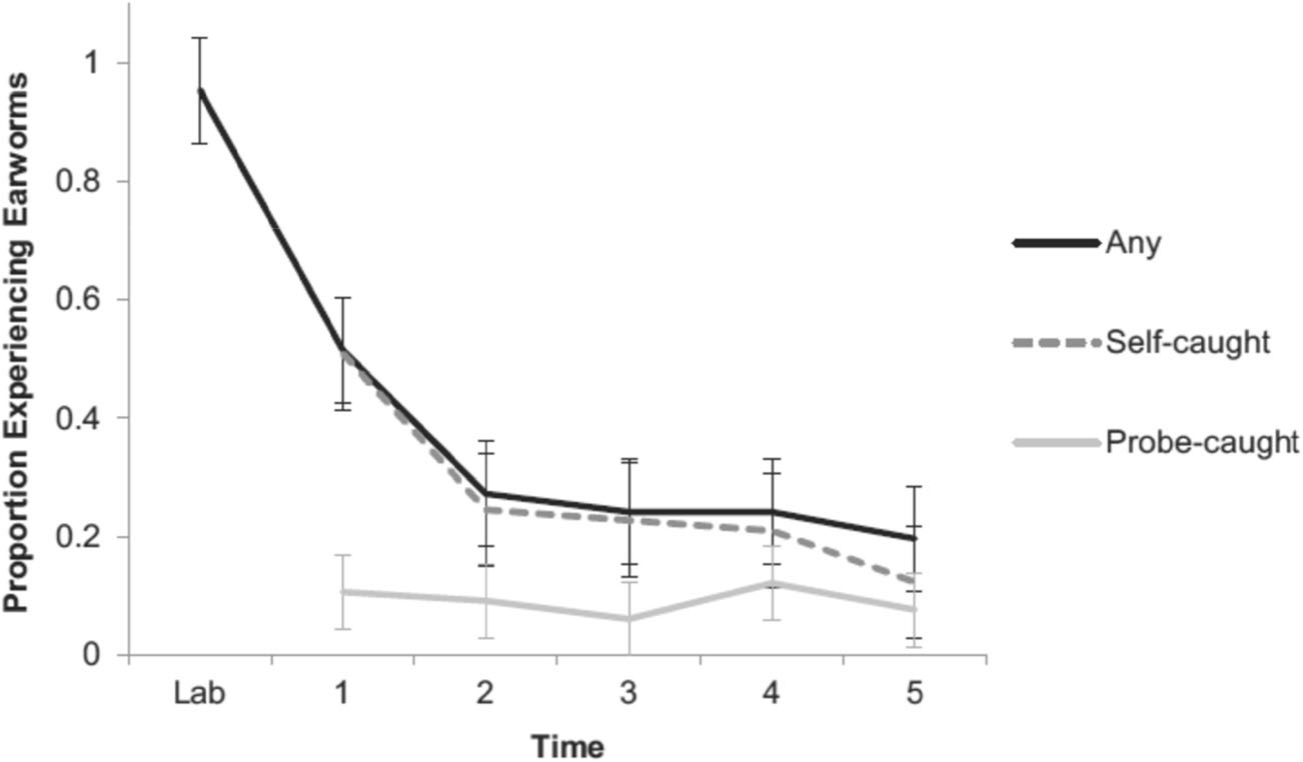
Overview of INMI induction success across sampling points in the study of Moeck et al. (2018). Reproduced with permission, © American Psychological Association

#### Subjective pleasantness

Research shows that the subjective assessment of INMI pleasantness depends on at least two factors: the features of the experience and the mode of inquiry. Several studies indicate that increasing repetition of music increases negative appraisal (Floridou & Müllensiefen, 2015; Williamson & Jilka, 2014). A study focused on INMI intrusiveness (Hyman et al., 2015) concluded that the defining factors for negative, intrusive experiences included the involvement of disliked music and excessive looping of the piece. For some unknown reason, less known and less liked songs were perceived to loop more often. One ESM study also found that difficulties in identifying INMI triggers (see the Dynamics of INMI section) reduce INMI pleasantness (Floridou & Müllensiefen, 2015).

However, when specifically probed, people consistently appraise INMI as a generally positive or neutral experience. In a sample of 190 undergraduates, preferred music was self-reported as musical imagery almost thrice as often as aversive music (Beaty et al., 2013). Similar results have been found in retrospective (Halpern & Bartlett, 2011; Liikkanen, 2012c), diary (Beaman & Williams, 2010; Halpern & Bartlett, 2011), and ESM studies (Floridou & Müllensiefen, 2015) of INMI. Floridou and Müllensiefen (2015) associated the highest probabilities of a pleasant experience with specific, identifiable memory triggers (.67), sound association (.50), recent musical exposure (.41), and contemplation (.38). Another indicator of the favorable nature of INMI comes from the finding that participants in a diary study reported that INMI did not interfere in their activities 66% of the time (Beaman & Williams, 2010).

Subjective pleasantness of INMI can also be indirectly assessed using sentiment analysis. Liikkanen et al.’s (2015) study of Twitter data compared the emotional valence of tweets about INMI against both musical and nonmusical reference data and found that Twitter users discussed INMI experiences more frequently in a negative tone. This indicates that people may be biased toward reporting their more bothersome INMI experiences openly on social media.

#### Phenomenology of INMI: Discussion

Perhaps the main concern regarding INMI research is construct validity, which based on the reviewed data is best justified by two lines of work: Replicated findings and the empirical results on INMI veridicality. The key replicated finding is that INMI experiences are found in both probed and spontaneous reports across the world (Liikkanen, 2012c; Liikkanen et al., 2015; Williamson et al., 2012). This adds credence to the claim that self-reporting can, with known biases, be trusted as a data source. For instance, it is now understood that retrospective, ESM, and concurrent reporting can each generate different estimates of INMI duration. “Retrospective meta-memory for involuntary semantic memories may be less accurate than measures obtained from experience sampling”, conclude Floridou and Müllensiefen (2015, p. 479).^2^ Further work is needed to develop methods that accurately and unobtrusively measure the duration of INMI as it occurs (for instance, following the initial efforts of Moeck et al., 2018).

The studies of musical imagery veridicality provide another type of evidence on the validity of the experience. These studies show high fidelity for melody and tempo for INMI. For instance, INMI songs are typically imagined within 15% of the canonical tempo (Jakubowski et al., 2015). Findings on INMI are congruent with what has been found in the studies of voluntary imagery, providing evidence that involuntary and voluntary musical imagery experiences operate via the same underlying mechanisms (Jakubowski et al., 2018). Future work should probe the veridicality of other musical features, such as timbre and texture, to provide evidence as to whether certain elements of music may *not* be faithfully reproduced within INMI (in line with the suggestions of Bailes, 2007).

In sum, studies of the phenomenological aspects of INMI have revealed that INMI is widespread and frequent in everyday life. According to ESM studies, people experience INMI in the range of 17%–47% of the time when contacted, effectively explaining retrospective reports in which more than 90% of respondents report INMI on at least a weekly basis. When directly asked, most of the time people are fond of their INMI experiences and mostly experience preferred music, although unprompted reports reveal that INMI can be intrusive.

### Dynamics of INMI

The dynamics of INMI theme entails studying the conditions that promote or hinder INMI, including situational factors. This work began by validating methods for INMI induction (i.e., intentionally evoking INMI experiences using experimental manipulations under at least quasicontrolled circumstances). Other studies included within this theme have presented self-report data on the triggers that precede INMI experiences and the coping strategies people employ to eradicate unwanted INMI.

#### Awareness of INMI triggers

INMI experiences often have recognizable triggers (Jakubowski et al., 2015; Williamson et al., 2012; Williamson, Liikkanen, Jakubowski, & Stewart, 2014). The pioneering study on this topic (Williamson et al., 2012; Williamson & Müllensiefen, 2012) utilized a grounded theory approach to analyze 604 free-form reports about the circumstances preceding a recent INMI experience. The most prominent of the four main themes that emerged was musical exposure, which was divided into recent and repeated forms. In addition, several types of memory triggers were identified, with different forms of association being the most typical. An example of an association is, “Every time I travel along the same road in Blackpool” (Williamson et al., 2012, p. 271). The affective-states theme referenced mood, stress, and surprises as the antecedents of INMI episodes. The final low-attention-states theme described situations in which a person has very little on their mind. Williamson and Müllensiefen (2012) concluded that “there are a number of non-musical stimuli in the environment that can trigger the memory of a musical excerpt” (p. 1127), indicating the multimodal and cross-modal connectivity of the musical memory system.

One diary study found that participants were able to identify the trigger of their INMI experience 72% of the time (Jakubowski et al., 2015), whereas another diary study produced a somewhat lower figure of 56% (Halpern & Bartlett, 2011), and an ESM study reported a figure in between these two estimates of 62% (Floridou & Müllensiefen, 2015). A study involving an optional probe of potential reasons for an INMI episode showed that 148 out of 648 reports (22.8%) included participant input on the matter (Byron & Fowles, 2015). Recent exposure, memory triggers, inherent catchiness of the music, and repeated exposure were repeatedly nominated by the participants as possible reasons for their INMI experience.

#### Experimental induction of INMI

Eight publications from eight research groups worldwide have shown that experimental induction of INMI is possible (see Table 1). This has been demonstrated using data collected in a combination of settings, including online, laboratory, and naturalistic paradigms. In an online study, Liikkanen (2012a) utilized a familiarity-controlling lyrics completion task to prime and trigger INMI during a subsequent form completion task. Subsequent studies have utilized laboratory conditions, at least to initially expose participants to potential INMI songs. At times, the outcome has been reported outside the laboratory, in the natural environment. For example, Byron and Fowles (2015) chose two previously unknown tracks and played them to participants in the laboratory either two or six times, to create low and high familiarity conditions. In the following 3-day period, participants were contacted approximately every 2 hours to complete experience sampling reports (18 in total). The initial INMI induction rate of 80% decreased exponentially over the 3 days, and music from the high familiarity condition was experienced much more frequently. Similarly, in a study using a combination of filler and thought-monitoring tasks (Moeck et al., 2018), an immediate INMI induction rate of 94% was achieved, which decreased significantly over the 8 hours following initial musical exposure, as measured via ESM (see Fig. 7). Finally, Floridou et al. (2018; Floridou et al. 2017) used a covert induction paradigm to investigate the occurrence of repetitive INMI. Repetitive INMI were reported in 58% of the immediate (5-min delay) reports and in 50% of the reports on the following day (Floridou et al. 2018).

**Table 1:** List of INMI induction studies with their details specifying number of participants, stimuli, measurement interval, sampling type, and success rate

Publication	N part.	Number of stimuli	Measurement interval	Sampling type	Success rate
Liikkanen, 2012a	991 + 6524	5	Few minutes	Retrospective	68% + 50%
Hyman et al., 2013	89 +139 + 123	18 + 3 + 3	Days + 10 min + 10 min	Retrospective	55% + 10%–55%
Beaman, Powell, & Rapley, 2015	44 + 18 + 36	1	3 min	Concurrent	100% + 100% + 95%
Byron & Fowles, 2015	36	2	3 days	ESM; 2-hourly	36%
McCullough Campbell & Margulis, 2015	120	1	5 min	Retrospective	67%–87%
Floridou et al., 2017	200	2	5 min + 24 h	Retrospective	60%–65%
Floridou, Williamson, & Emerson, 2018	60	2	5 min + 24 h	Retrospective	68% + 59%
Moeck et al., 2018	143	5	5 min + 8 h	Retrospective, ESM	94% + 62%

Several studies have revealed insights on how aspects of the presentation of particular musical stimuli affect subsequent INMI induction. It has been found that short music samples are adequate for INMI induction, as shown by a paradigm in which a 30-second extract of a single track was played twice (Beaman et al., 2015). This paper reported systematic INMI induction success following a thought-suppression experiment. A series of studies conducted by Hyman et al. (2013) was partially motivated by the search for the Zeigarnik effect, defined as “unfinished activities and thoughts staying active in memory and consciousness longer than completed thoughts” (p. 207). The rationale is that unfinished tasks can remain accessible in metamemory until the tasks are completed. The researchers only found partial support for the Zeigarnik effect—songs that were experienced as INMI immediately following the induction procedure were more likely to resurface later as INMI, however songs that were initially presented in incomplete versions were *not* experienced more frequently as INMI than songs presented in their entirety. It was also found that songs that were well known to the student participants were reported as INMI more than three times more often than unfamiliar songs (55% vs. 17%, respectively; Hyman et al., 2013, Study 2).

#### Interactions with working memory load, attention, and movement

Studies conducted within the cognitive psychology paradigm commonly make reference to key cognitive capacities, such as attention and working memory, to explain INMI. Floridou and Müllensiefen (2015) found that mind wandering (including INMI) is more likely when people feel drowsy, lonely, tired, bored, or are undertaking an activity with low cognitive load. This corroborates earlier studies which have implied that INMI is more likely to occur under sensory deprivation, during idle moments, or when tired or bored (Liikkanen, 2012c). Some activities seem to be more likely to coincide with INMI, such as commuting, exercising, or working (Liikkanen, 2012c). Bailes (2015) showed that the likelihood of imagining music (voluntarily or involuntarily) was highest between 11:00 a.m. and 1:00 p.m. in her ESM study, but other evidence (Byron & Fowles, 2015; Floridou & Müllensiefen, 2015) suggests that the time of day is meaningless without considering mediating activities. These studies are based solely on self-reporting in relatively uncontrolled settings, and experimental research has been able to further clarify the effect of situational demands on INMI onset.

Three experiments by Hyman et al. (2013) utilized a priming procedure with three songs (contemporary pop or Beatles songs). After listening and simultaneously rating the song, participants completed a 5-minute task that varied in type and difficulty across experiments. They subsequently completed a questionnaire on their INMI experiences during the task. In the visual maze task, the authors witnessed a 75% INMI induction success rate, in the visual/arithmetic Sudoku task the success rate was below 50%, and in the verbal anagram task it was less than 40%. The study thus indicated that task type had a prominent effect, which the researchers attributed to different components of working memory being required in the tasks and conflicting with the onset of INMI. However, counterintuitively, the participants reported fewer INMI during an easier task than during the more difficult one. The number of manipulations used in this study was too few to investigate any potential quadratic relationship—for instance, whether a medium-difficulty task resulted in fewer INMI than both an easy and difficult task.

Recently, the impact of cognitive load on INMI was investigated in an induction study using a dot-counting task (Floridou et al., 2017). The task varied in difficulty, introducing three levels of cognitive load. The authors found that the INMI induction success rate systematically decreased as cognitive load increased (33%, 25%, and 20% corresponding to low, medium, and high cognitive load tasks). This finding stands in contrast to Hyman et al.’s (2013) reversed trend.

INMI also appears to have a connection to body movement. In work by McCullough Campbell and Margulis (2015), 120 participants heard a song and were instructed to either listen, move to, or sing to the music. INMI induction success rates ranged from 45% to 63% (during a subsequent distraction task) and 60% and 87% (throughout the experiment). Induction success was dependent on the conditions: participants who moved and vocalized showed the highest INMI levels across both measurement points. The facilitating effect of movement is notable, given that another empirical study reported that 25% of INMI episodes occurred during a repetitive movement in everyday life (Jakubowski et al., 2015).

Finally, a series of experiments aimed at testing a potential strategy for reducing the occurrence of INMI was conducted by Beaman et al. (2015). They tested whether simple activation of the vocal apparatus by chewing gum might reduce INMI. The motivating hypothesis was that irrelevant subvocalization would compromise recall by degrading the quality of the verbal memory representation, which has previously been proposed in the subvocalization theory of auditory imagery (Smith, Reisberg, & Wilson, 1992). The studies used a paradigm in which the participants were first primed with a single song. The results from the following thought-suppression experiment (“try not to think of the song you just heard”) revealed that chewing gum decreased the frequency of INMI reports. The suppression effect was notable (η^2^ = .18), and the gum-chewing task resulted in significantly fewer INMI episodes than a similarly motor intensive task of finger tapping. The study thus pinpoints the specific role of articulatory motor activity in both the voluntary and involuntary mental recollection of music (Beaman et al., 2015).

#### Influence of competing stimuli

What factors influence the likelihood that one song will be experienced as INMI compared with another? Recency and primacy effects are well documented in working memory research (Baddeley & Hitch, 1993; Davelaar, Goshen-Gottstein, Ashkenazi, Haarmann, & Usher, 2005). The first demonstration of an INMI recency effect was reported in a study that presented five stimulus songs in a counterbalanced order across five experimental groups and found an elevated probability for the final stimulus song to occur as INMI (Liikkanen, 2012a). Other induction studies have since corroborated the existence of the recency effect (Byron & Fowles, 2015; Hyman et al., 2013). The recency effect was particularly clear in a series of three experiments (Hyman et al., 2013), in which the third, final song consistently showed a greater likelihood of being reported as INMI. None of the studies found evidence for the primacy effect.

A study comparing contemporary pop songs with older, but well-known popular music showed that contemporary songs produced an 18%-unit higher induction success rate (Liikkanen, 2012a). However, a series of controlled laboratory studies found no difference in the success rate between songs by The Beatles and contemporary pop music (Hyman et al., 2013). Liikkanen’s (2012a) experiments also demonstrated that the same stimulus song presented among different competing stimuli had a remarkably different likelihood of being experienced as INMI (9% vs. 51%). In addition, one study has investigated the impact of the emotional valence of musical stimuli on INMI induction rate, but found no effect in this regard (Moeck et al., 2018).

#### Coping with INMI

Several studies reveal that people take both precautionary and reactive actions to reduce or eradicate unwanted INMI (Beaman & Williams, 2010; Liikkanen, 2012c; Williamson et al., 2014). For instance, Beaman and Williams (2010) reported that their survey and diary respondents most often used two displacement strategies: general displacement and specific (musical) displacement. An example of general displacement is focusing on work, while an example of specific displacement is thinking of another song. However, the data revealed that displacement strategies predicted a worse outcome (longer INMI duration) in comparison with doing nothing. The authors relate this finding to the ironic mental control theory, which postulates that attempts to monitor the contents of consciousness to block certain thoughts can be counterproductive (Wegner, 1994).

Coping strategies were further investigated in a study comparing INMI experiences drawn from the UK and Finland (Williamson et al., 2014). This study demonstrated that respondents across different geographical and language regions employ variable and intricate, but similar, coping tactics. These predominantly included the same displacement strategies as those reported by Beaman and Williams (2010), with greater variation in musical and nonmusical displacement strategies. Interestingly, Williamson et al. (2014) found that most of the time, people do not actively try to inhibit INMI, but “let it be.” Many respondents also described good mental control over their INMI.

#### Dynamics of INMI: Discussion

A methodological leap in developing experimental setups that enable the induction of INMI has permitted researchers to study its dynamics, by manipulating factors that might influence the onset and perseverance of INMI in systematic ways. Eight studies from the review recorded successful INMI induction procedures, with success rates ranging from 36% to 100%, and a median of more than 60%. These studies collectively show that recent and repeated exposure to music primes our musical memory for the later emergence of INMI, which is compatible with the long-term spreading activation account of involuntary memory (Kvavilashvili & Mandler, 2004). On the other hand, the similarities to other involuntary semantic memories fall short, as people are able to report a trigger that initiated their INMI the majority of the time (cf. identifiable triggers reported for 20% of involuntary semantic memories in Kvavilashvili & Mandler, 2004). However, as all studies cited here have used self-report methods to identify triggers, one question that remains is the degree to which participants are *accurate* in identifying such triggers. In addition, a recency effect has been revealed, indicating that if several song traces are activated in memory within a short time window, the last one of these is most likely to be experienced as INMI later on.

**Fig. 8:**
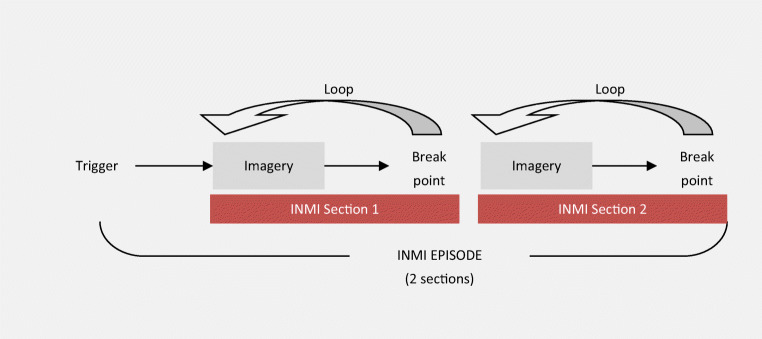
Internal structure of an INMI episode, consisting of triggered imagery of one or more sections (two sections exemplified here). Sections may comprise imagery from the same or different pieces of music

Research on INMI dynamics facilitates our understanding of how an INMI experience unfolds. We have derived a schematic model, illustrated in Fig. 8, which describes the different parts of the INMI experience as a process. The illustration of the process begins with an internal (thought or feeling) or external (perceived stimulus) trigger, which initiates the imagery process. The imagery proceeds, possibly over only a few measures of music, until an ill-defined breakpoint, at which point a single INMI section has concluded and the imagery loops back to the starting point, possibly with a delay (nonrepetitive instances of INMI; i.e., musical mind pops, do not include this loop component). A singular INMI episode can comprise one or more sections of music, each of which may or may not loop, experienced as an uninterrupted stream of mental music.

Experimental studies employing secondary distractor tasks have found that the onset of INMI is sensitive to situational demands. Several studies suggest that low-attention states and idleness are common precursors to INMI (Floridou & Müllensiefen, 2015; Liikkanen, 2012c; Williamson et al., 2012), while a smaller body of evidence has indicated that INMI frequency decreases during tasks high in cognitive load (Floridou et al., 2017) and particularly in verbal tasks (Hyman et al., 2013). Beyond strictly mental activities, INMI shows an interesting and not yet well understood relationship with bodily movement and the activation of vocal articulators. Studies have found that requiring participants to move to and vocalize songs renders them more prone to INMI induction (McCullough Campbell & Margulis, 2015), whereas when articulation is blocked by chewing gum, fewer INMI episodes are reported (Beaman et al., 2015).

INMI does not always remain in the background, and at times it can be a source of frustration. This leads to diverse coping actions. Displacement strategies, with and without music, are the most typical (Beaman & Williams, 2010; Williamson et al., 2014). However, data on the actual efficacy of different coping strategies are too scarce to present conclusions about their applicability. Overall, evidence shows that the ironic character of thought (Wegner, 1994) may hinder attempts to control INMI and the conscious effort to suppress imagery may evoke self-monitoring, which leads to sustained episodes of INMI (Müllensiefen et al., 2014). Countermeasures that rely on automated, nearly passive motor activity, such as chewing gum (Beaman et al., 2015), could be helpful, but their usefulness remains to be verified in naturalistic settings.

Experimental controls do not always work perfectly. This has been observed both in studies that attempted to manipulate participants’ responses to music (McCullough Campbell & Margulis, 2015) and in research on cognitive load. Floridou et al. (2017) recounted difficulties in both maintaining consistent load and ensuring that participants followed the manipulation as instructed; this problem also appears to have negatively affected the experimental setup of Hyman et al. (2013). This leads to the suggestion of using several tasks of varying complexity and constant load, which could mitigate against wavering attention and rising boredom.

### Individual differences in INMI

Virtually all aspects of INMI phenomenology are subject to notable individual variation (Beaman & Williams, 2010; McNally-Gagnon, 2016; Williamson et al., 2012). Correlations between predictors and INMI phenomenology measures tend to be weak or moderate at best, indicating considerable residual variance, at least partially attributable to individual differences. However, studies do show that several factors systematically influence these differences. Factors such as gender, age, musical education and activity, and personality have been investigated in relation to INMI. It has also been demonstrated that people reporting subjective differences in the frequency and appraisal of INMI exhibit differences in cortical structure (Farrugia et al., 2015), indicating that these behavioral differences can be manifested at a biological level.

#### Age and gender effects

The strongest evidence supporting a gender effect (Liikkanen, 2012c) is the higher proportion of women reporting INMI several times a day (27% vs. 24%; women vs. men; see Fig. 5). Since then, few studies have tried to or have possessed adequate power (Beaman & Williams, 2010, 2013; Hyman et al., 2013) to replicate this finding. Two experimental studies to induce INMI found that female participants reported more INMI episodes (Liikkanen, 2012a; McCullough Campbell & Margulis, 2015), although another induction study showed no such difference (Moeck et al., 2018).

A weak negative relationship between age and INMI frequency has also been found (see Bennett, 2003; Liikkanen, 2012c), but this result has not been consistently replicated. For instance, Bailes (2015) found a positive relationship between age and the number of musical imagery episodes (including both voluntary and involuntary imagery) reported in an ESM study. However, she attributed this to the mediating factor of musical practice, which also had a strong relationship to INMI frequency in Liikkanen’s (2012c) study. Another study showed a negative, marginally significant association between age and interference of INMI (Beaman & Williams, 2010).

#### INMI-prone personalities

Personality traits, as measured by standard instruments, have shown associations with various facets of self-reported INMI. Using the Obsessive-Compulsive Inventory–Revised (OCI-R; Foa et al., 2002) in a study of 1,536 participants, Müllensiefen et al. (2014) found that subclinical obsessive-compulsive (OC) traits were positively related to self-reported INMI disturbance and frequency, and were indirectly related to INMI duration and unpleasantness (mediated by INMI disturbance). In developing the IMIS instrument, Floridou et al. (2015) demonstrated that multiple subscales of a daydreaming and mind wandering scale (Imaginal Processes Inventory, IPI; Singer & Antrobus, 1966) correlated weakly, but positively with INMI frequency.

The Big Five personality traits of openness to experience and neuroticism have shown positive relationships to INMI experiences across several studies, with variable degrees of strength, although at a consistently significant level (Beaman & Williams, 2013; Beaty et al., 2013; Cotter et al., 2016; Floridou, Williamson, & Müllensiefen, 2012; Kellaris, 2001). Some of these studies have revealed relationships with INMI frequency, such as Cotter et al. (2016), who found a positive correlation between INMI frequency and openness to experience (measured via the NEO-PI-3; Costa & McCrae, 2010), which was attributable to the Fantasy and Aesthetics subscales (*r* = .31 and *r* = .24, *p* < .001). Other studies have reported no significant relationships between personality factors and INMI frequency, but found neuroticism and openness to be positively related to other aspects of INMI phenomenology, such as INMI duration (Floridou et al., 2012). Some of this variation in results may potentially be attributed to differences in the scales used to measure both personality and facets of INMI between studies.

The study by Cotter et al. (2016) also examined the relationship between responses on the IMIS and a schizotypy instrument (Wisconsin Schizotypy Scale; Winterstein et al., 2011). Schizotypy indexes subclinical schizophrenia spectrum symptoms, including both positive (e.g., increased presence of magical ideation, distorted perceptions, hallucinations) and negative (e.g., diminished pleasure and motivation) symptoms. The authors hypothesized that individuals high in schizotypy would be more likely to experience INMI. Statistical associations between the schizotypy scale and INMI frequency were relatively weak, but statistically significant (*r* = .17, for magical ideation; *r* = .19, for perceptual aberration).

Schizotypy was also the main interest in a study of 127 UK students (Beaman & Williams, 2013). The study used the Schizotypal Personality Questionnaire (SPQ; Raine, 1991) together with a thought-suppression scale, the White Bear Suppression Inventory (WBSI; Wegner & Zanakos, 1994), and a custom INMI survey. Schizotypy was positively associated with worrying over INMI, the degree to which INMI prevented daily activities, difficulty in dismissing INMI, and INMI duration. These correlations ranged from .326 to .388. The study also found an association between the mental suppression and intrusion scales of the WBSI and several INMI items (length, difficulty in dismissing, disruptiveness, and interference), with the degree of association ranging from *r* = .18 to .37. The authors note that although the SPQ and WBSI are correlated, both have some unique explanatory power in predicting the intensity of INMI experiences.

Finally, a relevant question is whether some people are more sensitive to inner sensations/thoughts and thereby more susceptible to INMI than others. Two studies have investigated the role of transliminality (Lange, Thalbourne, Houran, & Storm, 2000), a measure of such sensitivity, and its relationship to general musical imagery and INMI frequency, respectively (Bailes, 2015; Baruss & Wammes, 2009). These studies found some support for this hypothesis, albeit via relatively small correlations (*r* = .25 and *r* = .27, respectively) and sample sizes (*N* = 47 and *N* = 67, respectively).

#### Musical training and engagement

Several studies have found differences in INMI phenomenology in relation to musical training. It has been reported that music students experience more INMI than other students (Beaty et al., 2013; Hyman et al., 2013). Liikkanen’s (2012c) results indicate that musicians experience more instrumental and novel music, and that active musical practice predicts more frequent INMI. A survey with a substantially smaller sample (Hyman et al., 2013) found that musicians had more frequent intrusive INMI experiences, as well as more recent and detailed INMI experiences, than nonmusicians did. In addition, musicians’ INMI was reported as 71% longer in duration than nonmusicians’ INMI in the context of an induction study (Moeck et al., 2018).

The evidence is equivocal, however, as not all studies have found differences in INMI frequency related to musicianship (Beaman & Williams, 2010; McNally-Gagnon, 2016; Müllensiefen et al., 2014) or have produced mixed or negative results (Floridou et al., 2015). For instance, in one induction study, music students did not show increased INMI susceptibility, whereas frequent music listening did have a positive effect (McCullough Campbell & Margulis, 2015).

INMI appears to be associated not only with formal musical training but also with several types of engagement with music. Active engagement with music favors the later emergence of INMI (Beaty et al., 2013; Liikkanen, 2012c). A survey study (Liikkanen, 2012c) demonstrated that musical practice and music listening were the strongest predictors of self-reported INMI frequency. Another study has suggested that singing alone is positively associated with INMI frequency (Williamson & Müllensiefen, 2012). The frequency and type of musical engagement have been demonstrated to play a role both qualitatively as well as quantitatively in INMI experiences (Filippidi & Timmers, 2017). According to this diary and survey study, more pleasant INMI experiences are associated with frequent and functional uses of music.

One study (Weir, Williamson, & Müllensiefen, 2015) has explored whether there is a relationship in the reverse direction—that is, whether INMI frequency might influence perceptual or performative musical skills. This study compared two groups of participants, who reported typically experiencing either a high or low frequency of INMI, in their performance on a voluntary musical imagery task involving pitch and timing judgments. Although musical training had a positive effect on participants’ performance on the pitch imagery task, the INMI measures did not predict success on either task, suggesting that frequent INMI experiences do not improve voluntary musical imagery skills, at least in terms of pitch or temporal acuity.

#### INMI in comparison to other types of involuntary memories

People commonly report other types of involuntary semantic memories; however, INMI appears to be the most of prevalent of these. Specifically, Liikkanen (2012c) compared INMI with involuntary visual, word/sentence, tactile, olfactory, kinetic, and motor imagery, and found music to be the most often encountered involuntary memory. Hyman et al. (2015) contrasted INMI with images, (autobiographical) memories, and thoughts related to the future, romance, friends, work, and money. The resulting weak positive correlations demonstrated that those who have frequent INMI episodes also tend to notice involuntary memories of other types.

According to a survey study involving 44 people with musical hallucinations and 226 participants without hallucinations (Moseley et al., 2018), the characteristics of INMI are distinct from those of mental disorders such as musical hallucinations. It was found that INMI experiences are more frequent, more controllable, more lyrical, and more familiar than hallucinations. Hallucinations are also not as repetitive as INMI experiences, making them different on several phenomenological aspects and challenging the proposition that musical hallucinations are an extreme variation of INMI (Liikkanen, 2012b).

#### Individual differences in INMI: Discussion

Individual differences in INMI are considerable. They most prominently relate to musical activities that, particularly in the long term, may influence INMI experiences both qualitatively and quantitatively. For instance, musical training may extend the duration of INMI episodes almost threefold. The causal factor may be some yet undiscovered aspect of musicality manifesting in both overt and covert musical life. However, this has not been evidenced in tasks requiring deliberate recall of low-level features of music such as pitch or tempo (Weir et al., 2015). A few studies have indicated that women may be more susceptible to INMI than men are (e.g., Liikkanen, 2012a, 2012c). However, the gender effect is not large, and it has been suggested that gender should be treated as a nonbinary variable, which could help reduce variance (Floridou, 2015). Finally, personality measures relate to self-reported INMI experiences. The correlations between trait factors and INMI phenomenology have been significant across several studies, although never particularly strong. In particular, studies have repeatedly shown connections between the Big Five factors of “openness to experience” and “neuroticism” and INMI phenomenology (Beaman & Williams, 2013; Beaty et al., 2013; Cotter et al., 2016; Floridou, Williamson, & Müllensiefen, 2012; Kellaris, 2001).

One topic in need of further investigation is whether aging influences INMI experiences, as both voluntary and involuntary memory recall typically decrease with age (Schlagman, Kliegel, Schulz, & Kvavilashvili, 2009). The results to date on INMI and aging are weak and somewhat contradictory. To fully understand if features of INMI change across the life span, developmental research is also required, as essentially all INMI research to date has focused on adult populations. This means all questions regarding the emergence of INMI in childhood remain unexplored.

### Musical features of INMI

#### Importance of familiarity, preference, chorus, and lyrics

Idiosyncrasy best describes the spectrum of pieces of music people report as INMI. The repeated finding across studies has been that the repertoire of INMI music is extremely variable, and specific pieces of music show little repetition between or within individuals (Beaman & Williams, 2010; Jakubowski et al., 2015; Liikkanen, 2012a), unless the researchers specifically manipulate song exposure. In addition, research participants have reported INMI experiences for a wide variety of styles of music, such as pop, classical, jazz, children’s songs, TV jingles, and film music (Beaman & Williams, 2010; Halpern & Bartlett, 2011; Hyman et al., 2013; Jakubowski et al., 2015). Despite this variability, four features are common across INMI experiences: high familiarity, liking the experienced music, locus around the chorus, and the inclusion of lyrics.

Several studies support the idea that familiar, better memorized music is more likely to be experienced as INMI. Recognizable, familiar music is systematically reported (Beaman & Williams, 2010; Halpern & Bartlett, 2011; Hyman et al., 2015). This is best illustrated by the fact that participants can, with very few exceptions, identify and name the tunes they experience as INMI. It has also been shown that when musical exposure is experimentally controlled, familiarity has a reliable effect on increasing the incidence of INMI (Byron & Fowles, 2015). However, some empirical studies have failed to find an effect of familiarity (Moeck et al., 2018) and instead argue that familiarity effects might be due to differences in catchiness. Several studies also show that liked or preferred music is more likely to be reported as INMI than disliked music (Beaman & Williams, 2010; Hyman et al., 2015; Liikkanen, 2012c), which may be related to greater previous exposure to liked music.

However, even though familiar music is most commonly experienced, several studies have reported INMI experiences for previously unfamiliar music after initial exposure (Byron & Fowles, 2015; Williamson et al., 2012), as well as experiences of truly novel, self-created music (Floridou, 2015; Liikkanen, 2012c). In Liikkanen’s sample, 25% of women and 33% of men classified as musicians reported sometimes experiencing novel INMI content.

Several studies using different methodologies concur that the musical content of INMI experiences for popular music tends to be focused around the song’s chorus (Beaman & Williams, 2010; Halpern & Bartlett, 2011; Hyman et al., 2013; Liikkanen, 2012c; McCullough Campbell & Margulis, 2015). It has been reported that 90% of experimentally induced instances of INMI are fragments of the chorus (McCullough Campbell & Margulis, 2015), and one third of diary study reports have involved solely the chorus (Beaman & Williams, 2010). Lyrics are the most commonly mentioned musical feature of INMI (Halpern & Bartlett, 2011; Liikkanen, 2012c). For instance, 18 diary keepers reported experiencing INMI with lyrics 83% of the time (Halpern & Bartlett, 2011). In an experiment by Beaman (2018), lyrical music induced almost 50% more earworms than did instrumental music. Interestingly, the language of the lyrics does not seem to be important; the respondents to Liikkanen’s (2012c) survey reported as much imagery in a foreign, but familiar language as in their native language.

#### The properties of an earworm song

Popular music songwriters are known for their desire to create catchy melodies (Seabrook, 2015). A corresponding movement in musicology and music information retrieval studies has tried to establish a “hit song science,” but has so far produced meager results (Frieler, Jakubowski, & Müllensiefen, 2015; Pachet & Roy, 2008). Against this background, research on the properties of songs that are likely to become INMI looks both lucrative and challenging. The question of identifying an ideal earworm stimulus is important because it places the focus on musical content and objectively shared properties rather than on recency, preference, or any other idiosyncratic property.

Research on the importance of musical characteristics is still in its infancy. A recent study (Jakubowski, Finkel, Stewart, & Müllensiefen, 2017) compared the melodic features of songs commonly nominated as INMI (by at least three participants in an online survey) to similar, popular songs from the UK billboard charts. Musical data mining approaches were employed to discover any features that might differentiate INMI songs from other contemporary hits. It was found that INMI songs had “more common global melodic contours and less common average gradients between melodic turning points than non-INMI tunes” (Jakubowski et al., 2017, p. 122). In other words, “INMI music” typically combines melodic shapes that are familiar overall with some uncommon interval patterns. INMI tunes also displayed faster average tempi than non-INMI tunes. Importantly, these musical features added unique explanatory value, beyond the popularity and recency of the songs, in terms of their potential to become INMI.

#### Musical features of INMI: Discussion

The studies of common musical features in INMI experiences have accumulated the least empirical evidence of the four themes in this review. Most INMI research to date has emphasized the idiosyncratic nature of the experience, as evidenced by the fact that in naturalistic approaches, such as diary studies, most participants report an entirely different set of music as INMI in comparison with other participants. However, large-scale survey approaches can reveal commonalities between INMI tunes reported by multiple participants (Jakubowski et al., 2017), which aligns with the longstanding everyday notion—which has more recently appeared in the music psychological discourse (see, e.g., Burgoyne, Bountouridis, van Balen, & Honing, 2013)—that certain pieces of music are inherently “catchier” than others. Although research to date has revealed some melodic features that appear to increase the likelihood that a tune will become INMI, in addition to broader features such as the presence of lyrics and common focus around the chorus, there is still much work to be done in this area. For instance, future research should seek to determine how participant-level (e.g., personality, individual familiarity with a song) and stimulus-level (e.g., musical features, emotional expression) factors interactively contribute to the INMI experience. There is also a need to investigate additional features of the music beyond their melodic structure, such as whether particular lyrics or chord progressions increase the likelihood that a piece of music will be experienced as INMI.

## Conclusions and recommendations

This review has shown how psychological studies over the past 15 years have established a new research topic known as *involuntary musical imagery*, or INMI. We identified 33 publications reporting 47 studies contributing to this topic. These studies provide robust evidence on INMI phenomenology and dynamics of the experience in relation to the stimulus environment, with findings on how INMI experiences vary as a function of individual differences and musical features beginning to emerge. The picture of INMI drawn here is one of a universal psychological experience, with reliably detectable characteristics. Although most empirical studies suffer from biases, they bespeak the universal nature of involuntary music as a part of the normal musical mind. However, this experience is also accompanied by remarkable variability.

### What does INMI tell us about memory processes in general?

“Involuntary and intrusive thoughts may be a hallmark of conscious experience rather than a response to traumatic memories” writes Hyman (2013, p. 214). The outcomes of the present review show that INMI research is informative in terms of normal psychology. Certainly, INMI is well aligned with the three types of memory recall originally proposed by Ebbinghaus (1964): implicit, voluntary, and involuntary.

Research on INMI is most relevant to the study of involuntary memory in general. Involuntary memories have three varieties: ordinary involuntary memories, chained memories, and traumatic memories (Mace, 2007). According to Mace (2007), these occur either in everyday life, during the process of voluntary or involuntary recall, or as a feature of a psychiatric syndrome, respectively. Comparing these with INMI reveals some shared characteristics, and repetitive INMI in particular shows a very specific chaining effect—chaining of the same memory. Traumatic memories have no corresponding INMI type; the intrusive variations of INMI depend more on how a person reacts to INMI and how frequently they find it debilitating. Like Mace’s (2007) discussion on the differences between the retrieval properties of involuntary memory types, this review gives a reason to suspect that INMI portrays a retrieval type of its own. This type is frequently occurring, has both external and internal triggers that are typically identifiable, usually evokes little emotional response, and tends to repeat the same segment of remembered material, rather than evoking a chain of related memories—although the experience may be triggered via chaining.

The literature reviewed here reveals several parallels between INMI, which is often regarded as a type of involuntary semantic memory (Kvavilashvili & Mandler, 2004), and previous findings on involuntary autobiographical memories (Berntsen, 1998). Mace, Atkinson, Moeckel, and Torres (2011) found that involuntary and voluntary autobiographical memories reported by the same participants were judged to be equally accurate (via confidence ratings given by the participants and associates involved in the remembered event). Musical imagery research can provide an even more rigorous test of memory accuracy, since features of the recalled music can be compared with the original, recorded version. Research on this topic has revealed that involuntary and voluntary musical imagery were both recalled relatively accurately in comparison with the recorded version, and, crucially, the two memory types did not significantly differ in recall accuracy, at least in terms of musical tempo, the main focus of Jakubowski et al. (2018). Taken together, these findings indicate that involuntary and voluntary memories rely on the same long-term memory representation, and differ only in terms of retrieval mode.

In addition, Jakubowski et al., (2018) found that INMI seems to elicit an increased emotional response in comparison with voluntary musical imagery. This parallels previous findings that involuntary autobiographical memories have more impact on mood and elicit greater physical response in comparison with voluntary autobiographical memories (Berntsen & Hall, 2004). This may be due to the unanticipated nature of the recall implicated in involuntary memories. Finally, INMI episodes typically have identifiable triggers (Jakubowski et al., 2015), which parallels findings on involuntary autobiographical memories (Berntsen, 1996), but differs from results on other types of involuntary semantic memories (Kvavilashvili & Mandler, 2004). Similar to involuntary autobiographical memories (Berntsen, 1998), INMI appears to be more often triggered by external cues than internal cues; for example, recent hearing of a piece of music and environmental cues such as a person, word, sound, or visual scene are more frequently reported as INMI triggers than as mood states or mind wandering (Williamson et al., 2012).

The cognitive capacities upon which INMI builds have not yet been fully clarified. Several authors (Beaman & Williams, 2010; Hyman et al., 2013) discuss the relationship between INMI duration and short-term versus long-term memory capacity. They note how the imagery of long sections of a song arguably requires recall from long-term memory. Hyman et al. (2013) further argue that failure to retrieve a subsequent section of a song that is less well familiarized could result in what we have called a breakpoint (see Fig. 8), with the song looping back to the beginning of the known verse. However, this hypothesis still lacks empirical support.

Finally, INMI exhibits several known features of memory recall. One example is the recency effect found in multiple studies of INMI (Byron & Fowles, 2015; Hyman et al., 2013; Liikkanen, 2012a). Liikkanen’s (2012a) discussion considered prolonged activation (i.e., long-term recency) to have an effect on INMI. This was based on the idea of spreading activation in long-term memory networks (Kvavilashvili & Mandler, 2004). Müllensiefen et al. (2014) also discussed how recently activated memories become “primed to involuntary reactivation that is either spontaneous or linked by spreading activation” (p. 332). One interesting possible divergence between INMI and involuntary autobiographical memory concerns the distinction between associative and repetition priming. Some research suggests that involuntary autobiographical memory is more susceptible to associative priming (e.g., recent thoughts prime related, but different memories) than repetition priming is (e.g., recalling a memory primes recall of the same memory later on; Mace, 2005). INMI experiences appear to be more akin to repetition priming, such that hearing or imagining a song primes activation of the same song later on. However, research to date has not fully addressed the extent to which INMI might also invoke associative priming—for instance, whether INMI for a song might prime another song from the same artist or genre to subsequently appear as INMI.

### Biases in reviewed studies

Overall, empirical studies on INMI have shown notable variability in methods and research themes. Although this may be justified, given the early stage of the research and the collective and distributed nature of the work, some biases may impact the conclusions of this review.

Publication bias is evident when considering the material accumulated, found eligible, and included in this review. From our own experience, we believe numerous efforts have been made to induce INMI, but the number of failed attempts is unknown due to the lack of reporting of negative results. This unfortunate shortcoming limits our understanding of INMI dynamics, as our conclusions derive only from the successes, potentially distorting the perspectives on the volatility of the experience.

In terms of sampling bias, the situation is reasonable, at least in terms of the fact that most evidence on INMI is not restricted to student populations. The majority of studies (54%) involved samples that were not limited to university students. However, the majority of studies describing INMI phenomenology are based on participants living in “Western” countries. In terms of sampling coverage, statistically representative, although nonrandom, samples of INMI experiences have been collected in five nations: Australia (Byron & Fowles, 2015), India (Liikkanen et al., 2015), Finland (Liikkanen, 2012c), the United Kingdom (Williamson et al., 2012), and the United States (Hyman et al., 2013; Hyman et al., 2015; Liikkanen et al., 2015). The Twitter-based study (Liikkanen et al., 2015) was able to draw naturalistic data from users from 173 geographic regions (ISO 3166-1). However, as it focused on conversations held in English, it is still biased in that regard.

This situation means that INMI research is subject to WEIRD bias (Heinrich, Heine, & Norenzayan, 2010). This is a serious shortcoming for understanding INMI as a hypothetically general component of music cognition. But how severe is this bias? The current situation affords only speculation and motivates future research. Referring to the analysis by Heinrich et al. (2010), some mental capacities may have a strong “nurture” factor. For example, the disposition for perceiving a visual illusion may only develop under certain conditions. Could this apply to INMI, and what could these conditions be? Is the widespread prevalence of INMI among the studied populations related to cultural norms that restrict overt musical expression in Western adults, globally spread technology-mediated popular music culture, or some other factor?

### Recommendations for future work

As an emerging research area, there are still many unanswered questions regarding the nature and experience of INMI. We explore some of what we see to be the most promising of these questions in the next three subsections.

#### Why does INMI occur?

One of the regular questions of public interest around this topic is “Why do songs get stuck in our heads?” We see two variations of “why” questions related to INMI—an easy one and a more difficult one. The easier one concerns why an INMI experience occurs at a specific time. This is something research has been able to address, showing a clear stimulus-triggered INMI relationship, at least in the majority of cases. The more difficult “why,” the “ultimate question of INMI,” addresses why humans experience INMI at all.

One theory of involuntary memory emphasizes that consciousness is a functional feature of the human mind (Baars, Ramamurthy, & Franklin, 2007). In the case of involuntary memory, this functionality is less clear, and with repetitive INMI it is even more questionable. In fact, INMI is reminiscent of the infamous “auditory cheesecake” labeling of the musical faculty of the mind (Pinker, 1997; but see Pylyshyn, 2002). The functional perspective proposed for involuntary memory includes that it may be necessary for normal recall of episodic and declarative memory, or for temporal orientation and planning (Rasmussen & Berntsen, 2009). No similar functional explanation has been identified for INMI to date, although some initial hypotheses have been put forward.

Memory consolidation is one hypothesized functional role for INMI that arises in a few existing discussions (Floridou et al., 2015; Liikkanen, 2012a; McNally-Gagnon, 2016). The hypothesis is that repetitive INMI might be a conscious reflection of a continuous, ongoing, usually unconscious memory consolidation process, which imprints new musical memories into long-term memory. However, the actual conscious experience might still be epiphenomenal. This “no smoke without fire” account would mean that the absence of INMI might be a sign of dysfunctional memory or unsuccessful consolidation. This remains to be investigated, although anecdotally the authors are aware of individuals who appear to have normal perceptive and expressive musical capacities but no recognition of INMI, which contradicts this hypothesis. It would be worth investigating whether these people really lack INMI experiences, do not notice them, or engage in spontaneous singing or humming instead of covert mental concerts.

Mood regulation is an alternative, but not mutually exclusive, hypothesized function for INMI, which we articulate here as the idea that the experience of INMI would be instrumental to the maintenance of everyday mood and emotion. Evidence of intentional mood regulation, which is facilitated by recorded music technology, exists (Bishop, Karageorghis, & Loizou, 2007; Saarikallio & Erkkila, 2007; van Goethem & Sloboda, 2011), and thus it seems possible that INMI could be unconsciously performing a similar function. Some preliminary evidence for this account exists. For instance, Bailes (2015) found some congruence between mood ratings for the same music when experienced in heard and imagined versions, and Jakubowski et al. (2015; Jakubowski et al., 2018) found that INMI tempo correlates positively with ratings of subjective arousal in a similar way to perceived music.

Given the limited evidence to date, it is also entirely plausible that INMI is nonfunctional. Some accounts of involuntary autobiographical memory have outlined the possibility that such memories are merely coincidental or accidental occurrences that come to mind occasionally due to overlap between cues from the current situation and the remembered event. Evidence that voluntary and involuntary memories show phenomenological similarities does not provide definitive proof that both (or either of these) types are functional (Mace & Atkinson, 2009). More targeted research on the possible functions of INMI is needed before the nonfunctionality hypothesis is invalidated. We suggest this topic be approached from a variety of methodological angles—from introspective reports by participants on whether they are able to identify any functional role for INMI experiences to experimental manipulations targeting the main proposed functions (e.g., mood control).

A final key question is why repetition is so prevalent in the INMI experience. If the “persistent musical track” consisted of different individual songs, this could be conveniently explained as the memory chaining experienced with other types of involuntary memory (Mace, 2007). However, it is often not. Instead, the typical experience consists of a single song fragment repeating consistently, setting INMI apart from involuntary autobiographical memories. Why does looping occur? Why does the imagery stop and then rewind? Dynamic systems in mathematics exhibit stable limit cycles (i.e., oscillations that repeatedly loop back to their starting point). This framework might be useful for explaining, or at least describing, the repetition in INMI. These questions remind us that the INMI loop’s breakpoint and the detailed duration of INMI episodes remain underinvestigated. Some of the unanswered questions related to the loop are: Does the repetition conform to the musical meter? Are there regular pauses between the breakpoint and the jump to the beginning? Is the breakpoint always at the same spot within a single episode?

#### The motor connection

Several studies have linked motor activity, vocal system activity, or singing with INMI (Beaman et al., 2015; McCullough Campbell & Margulis, 2015; McNally-Gagnon, 2016; Müllensiefen et al., 2014). Survey and diary study respondents have also acknowledged a strong connection between INMI and overt movement (Floridou et al., 2015; Jakubowski et al., 2015). These findings show that active motor participation improves INMI induction success, songs that appear as INMI often comprise melodies that are easy to sing, and melody and song lyrics are the best preserved elements within the INMI experience.

The relationship between musical imagery and articulation and subvocalization is a currently underdeveloped area. Beaman et al.’s study (2015) was the first to reliably connect INMI to this topic. However, the history of subvocalization in psychological research (Sokolov, 1972) and schizophrenia research (Smith, 1992) is long. The latter goes so far as to suggest a subvocalization theory of verbal hallucination. Subvocalization, myogenic activity in laryngeal musculature and other parts of the vocal track, may be involved in the production of hallucinatory voices heard by schizophrenics. In contrast, in INMI, the musical content is usually familiar, and thus not a subject for explanation (i.e., it is a recall product, and thus the proposition that subvocalization correlates with the presence of INMI does not explain INMI). However, subvocalization could potentially be an important biosignal that communicates the presence of INMI without the need for verbal reporting, which relies on conscious monitoring and meta-awareness (see also Pruitt et al., 2019, for exploration of similar ideas in voluntary musical imagery). Such work could present a possible alternative to complicated and costly brain imaging paradigms for collecting objective evidence on INMI.

#### Nature or nurture?

One entirely unexplored aspect of INMI concerns its initial emergence in childhood. This is also important when considering the WEIRD bias, as we do not currently know if only specific cultural circumstances make us “vulnerable” to INMI. Psychological research on the development of musical skill spans several decades, and studies have recorded spontaneous musical behaviors from an early age (Dowling, 1982). This reveals several potential questions that INMI research could complement. Although age has been used as a covariate in some studies, INMI research on the whole has concerned only adults and teenagers. Research on the early childhood emergence of involuntary musical experiences and behaviors could be revealing in terms of the nature of INMI and may add a new chapter on its phenomenology. Any such work should span the age range of at least 1 to 7 years, based on one author’s personal experience, with the null hypothesis that both voluntary and involuntary musical imagery capacities emerge at the same time. If their timing proved different, this could also be informative to the nonfunctionality hypothesis of INMI.

### Call for rigor and repetition

We would like to make three further suggestions to improve the quality of future INMI research:

1. Documenting all details
2. Defining INMI consistently
3. Doing it again

Our first recommendation is to ensure detailed reporting of methods, samples, and stimuli. Carrying out meta-analysis of the results of different INMI induction studies may become feasible in the future, but only if commeasurable reporting practices are in place. In the studies included in the current review, this was not always the case.

Second, researchers must note which variety of involuntary mental music they are investigating (see Fig. 1). This is, in our opinion, a serious impediment for progress in the field because the lack of a clear definition introduces a new source of variance to the research, as the data already show. Specifically, previous research has not always been transparent in regard to whether repetitive “earworms” and/or nonrepetitive musical mind pops are being targeted. Given the early stage of research in this area, it seems wise to at least initially consider the possibility that they are different phenomena. Outside the INMI research field, it has been suggested that repeated involuntary memories should be called “intrusive” (Kvavilashvili & Schlagman, 2011). However, the present evidence demonstrates that repetitive INMI is seldom perceived as intrusive, and therefore we do not believe it should be labeled as such. In addition, the use of the term “involuntary” should be clearly defined and explained to research participants, in terms of whether it is being used to describe the initiation of the imagery episode, subsequent management of the experience, or both (see Cotter & Silvia, 2019). References to the model of the INMI process presented in Fig. 8 could be useful for defining and examining different components of the INMI experience.

Finally, replications of previous studies in different laboratories and cultural contexts would be extremely valuable. It is apparent from this review that many researchers have now successfully devised their own paradigms. Next, it would be important to attempt to reproduce these findings.

### Conclusions

The research evidence on INMI to date is far from parsimonious, but suggests the universality of the experience and demonstrates that it is a somewhat predictable and controllable phenomenon. For instance, individuals’ chosen behaviors, particularly musical immersion, can play a substantial role in defining how they experience INMI. This means that people’s intentional choices can have causal influences on how their INMI experiences manifest in the future. The combined results across the reviewed studies indicate that the occurrence of INMI can be predicted by a combination of factors attributed to the context/situation, individual experiencer, and musical stimulus itself. Future research should further explore the specific weightings and interactions of these influential factors.

The relatively short history of this research field means there are still a great many opportunities to explore the phenomenology of INMI, including a necessity for further cross-cultural and developmental research on the topic. New interventions inspired by the treatments for obsessive-compulsive disorder or other psychiatric diseases may reveal effective solutions to the challenging question of “how can you stop earworms?” Moreover, on the basis of current research, we do not even know if going entirely “earworm-free” is desirable at all, as the presumption that INMI is epiphenomenal has not been adequately addressed. For instance, the question as to whether INMI might play a functional role in everyday memory or mood processes is still highly underinvestigated. There is yet much to learn about the mind through the study of INMI, but the path laid out by the pioneering studies looks promising.

#### Author note

L.L. received a University of Helsinki internal grant to work on an early version of the manuscript. L.L. is grateful for the support of University of Helsinki and guidance of Mari Tervaniemi during the process leading up to this publication.

#### Open practices statement

No new data were collected for this article; the list of publications from which this review article draws is included in Supplementary Table 1. The work reported here was not preregistered.

## Electronic supplementary material

ESM 1 (DOCX 52 kb)

## Footnotes

1 It should be noted that the survey of Beaty et al. (2013) did not differentiate between voluntary and involuntary instances of musical imagery, and, as such, we cannot definitively conclude as to whether these results apply similarly to both imagery types.

2 Although absolute duration estimates may vary between these two methods, it should also be noted that Cotter and Silvia (2017) found a significant correlation (*r* = .56) between a retrospective INMI measure (IMIS) and an ESM measure of musical imagery (including both voluntary and involuntary imagery) in participant assessments of imagery episode length. However, no significant correlation (*r* = .11) was found for an analogous measure of imagery section length.
