# Supplementary material for: Involuntary musical imagery as a component of ordinary music cognition: A review of empirical evidence
Source: Psychon Bull Rev. 2020 Jun 24;27(6):1195–217. doi: 10.3758/s13423-020-01750-7 (PMC7704448; doi:10.3758/s13423-020-01750-7)
Supplement: Supplementary file 1 — (DOCX 52 kb) [file 13423_2020_1750_MOESM1_ESM.docx]

Supplementary Material

Supplementary Table 1. Detailed list of publications included in review, listed in order of publication from the earliest to the most recent. Details were collected from the publications. In the “Research theme” column, phenomenology is abbreviated as “Phen” and Individual differences is abbreviated as “Individual diff.” If the publication reports multiple studies, the number of participants (*N*) in each study is reported with a “+” delimiter. The same practice applies to whether the study sample consisted primarily of students, indicated by a “Y” or “N”, representing yes or no, respectively.

| **Citation** | **Research theme** | **Methods** | **Research questions** | **Main findings** | **N, Student** |
| --- | --- | --- | --- | --- | --- |
| Brown 2006 | Phen | Introspective case study | What is the experience of persistent INMI like? | Detailed description of INMI phenomenology in continuous experience. | 1, N |
| Bailes 2007 | Phen and Musical features | ESM study | What is the everyday musical imagery of music students like? | Common to report fragments of songs as musical imagery, associated with earlier exposure. | 11, Y |
| Baruss and Wammes 2009 | Phen and Individual diff | Survey | How can we quantify the characteristics of INMI? Does INMI relate to personality traits? | Introduction of Musical Imagery Questionnaire, report of an association between transliminality scale items and INMI persistence and distraction. | 67, N |
| Beaman and Williams 2010 | Phen and Musical features | Survey and diary study | What are everyday INMI experiences like? | INMI episodes are frequent, much of the experienced music is a repetition of a few familiar songs. Displacement strategies used for coping. | 103 + 12,  N + N |
| Halpern and Bartlett 2011 | Phen and Musical features | Survey and diary study | What characterizes INMI experiences? | INMI episodes are frequent, pleasant and linked to recent music exposure. Surveys and diaries correlate. | 18 + 41,  Y + Y |
| Liikkanen 2012a | Dynamics | Experimental study | Can INMI be experimentally induced? Does it exhibit serial position effects? | Induction succeeds with a variable rate depending upon the stimulus material. A small recency effect detected. | 991 + 6524 + 34, N+N+Y |
| Liikkanen 2012c | Phen and Musical features | Survey | Are INMI experiences common, what are they like, what influences them, how do they compare with other memories? | INMI is common in everyday life and its occurrence is influenced by several factors, particularly active music processing. | 11 910, N |
| Williamson et al. 2012 | Phen and Dynamics | Survey | Does the onset of INMI have recognizable characteristics? | Identification of four themes describing the initial conditions of INMI: Music exposure, memory triggers, affective states and low attention states. | 333 + 271,  N + N |
| Beaman and Williams 2013 | Phen and Individual diff | Survey | Are there differences in INMI with regards to personality? | Features of INMI found to correlate positively with dimensions of thought suppression and schizotypy. | 127,  Y |
| Beaty et al. 2013 | Phen and Individual diff | Survey + ESM | Does musical imagery differ with regards to musicianship or personality? How does sampling influence results? | Musical imagery is a positive and frequent phenomenon, particularly for musicians. Retrospective sampling creates bias. Openness to experience and neuroticism correlate positively with musical imagery. | 190 + 98,  Y + Y |
| Hyman et al. 2013 | Phen, Dynamics, and Musical features | Survey, experimental studies, and diary study | Does INMI display Zeigarnik or recency effects? Is INMI onset influenced by mental load? | Replicates earlier surveys, a strong recency effect found for successful priming, task difficulty determines the likelihood of INMI distraction, phonological task interferes more than a numeric one. | 299 + 16 + 89 + 139 + 123,  Y+ Y + Y+ Y + Y |
| Müllensiefen et al. 2014 | Phen and Individual diff | Survey | Do individual differences in obsessive-compulsive (OC) personality traits and behavior relate to differences in INMI experiences? | Singing and music listening influence INMI, subclinical OC is related to INMI frequency and perceived discomfort. | 1536, N |
| Williamson and Jilka 2014 | Phen and Musical features | Interview study | How are INMI experiences characterized, what form do they take and how do they make us feel? | Replication and elaboration of previous findings, with more detail. | 6, N |
| Williamson et al. 2014 | Dynamics | Survey | How do people react to INMI? Is this similar across countries and languages? | Reactions were similar across regions. Reactions ranged from acceptance to coping, such as distraction or engagement. | 1046, N |
| Beaman, Powell, and Rapley 2015 | Dynamics | Experimental study | Is INMI induction affected by suppressing subvocalization? | Chewing gum reduced musical imagery, effect attributable to motor suppression. | 44 + 18+ 36, Y + Y + Y |
| Byron and Fowles 2015 | Dynamics | Experimental study | Does familiarity with stimuli or levels of processing influence INMI induction? | More familiarity improved induction success, levels of processing did not. | 36, Y |
| Farrugia et al. 2015 | Individual diff | Brain imaging with MRI | Can correlates for the self-reported properties of INMI be found in brain structure? | Participants experiencing different responses to INMI showed variation in brain structures relevant to music processing. | 44, N |
| Floridou and Müllensiefen 2015 | Phen and Dynamics | ESM study | How frequent is INMI in everyday life, how is it perceived and how does it converge with activities? | INMI found in 47% of cases, influenced by the time of day. Triggers were usually identified and their cause influenced appraisal. | 38, N |
| Floridou et al. 2015 | Phen and Individual diff | Survey instrument development | Which properties of INMI can be reliably measured in research? | Presentation and initial validation of IMIS instrument to record INMI experiences. | 2646, N |
| Hyman et al. 2015 | Phen | Survey and experimental study | What are typical INMI experiences like? | Replication of earlier findings regarding phenomenology. | 293 + 15,  N + Y |
| Jakubowski et al. 2015 | Phen, Dynamics, and Musical features | Diary study | What is the temporal precision of INMI, and the influence of concurrent affective state on INMI? | Tempo of INMI corresponded well to recorded music. Imagined tempo was associated with arousal and valence. | 17, N |
| Liikkanen, Toivanen, and Jakubowski 2015 | Phen | Computational analysis of unprompted reports | Do people spontaneously share INMI experiences in social media? Do they describe INMI favorably? | Twitter users around the world discuss INMI. The discussions show a negative sentiment in comparison to general parlance. | 56 626, N |
| McCullough Campbell and Margulis 2015 | Dynamics | Experimental study | Do concurrent motor activities influence INMI induction? Do unexpectedly truncated melodies increase INMI? | Vocal and physical activation induced INMI more frequently. Melodic truncation does not affect INMI induction. | 120, Y |
| Weir, Williamson, and Müllensiefen 2015 | Individual diff | Experimental study | Do participants with frequent and persistent INMI differ in their ability to imagine musical pitch and tempo? | No association found between “extreme” INMI experiences and music perceptual skills. | 67, N |
| Cotter, Christensen, and Silvia 2016 | Phen and Individual diff | Survey study | Do aspects of INMI associate with personality traits? | Openness to experience, neuroticism, and schizotypy positively correlated with INMI frequency. | 182, Y |
| Floridou, Williamson, and Stewart 2017 | Dynamics | Experimental study | Does cognitive load influence INMI induction? Can INMI experiences be indirectly measured? | Increasing mental load decreased INMI probability. Indirect measures were possible and valid. | 200, Y |
| Filippidi and Timmers 2017 | Phen and Dynamics | Survey and diary study | What is the relationship of music listening and INMI experiences? | Listening conditions later INMI and INMI replaces music when not listening. | 329 + 11,  N + N |
| Jakubowski et al. 2017 | Musical features | Survey and computational analysis of INMI songs | Do INMI songs have specific structural properties? | Discovery of melodic features related to complexity and tempo that are associated with INMI songs. | - |
| Beaman 2018 | Theory and Dynamics | Review and experimental study | Do lyrical stimuli increase the likelihood of INMI experiences? | More INMI are reported with lyrical than instrumental stimuli and chewing gum reduced both instances. | 88, Y |
| Floridou, Williamson, and Emerson 2018 | Dynamics and Phen | Experimental study | How do different types of involuntary cognitions compare to INMI? | INMI experiences are more numerous immediately after induction, later on less negatively experienced and last longer. | 60, Y |
| Jakubowski et al. 2018 | Dynamics and Phen | ESM and diary | Are voluntary and involuntary versions of the same musical memory experientially similar? | Musical features, foremost tempi, correspond across recall types | 20, Y |
| Moeck, Hyman, and Takarangi 2018 | Dynamics and Phen | Experimental study | Does stimulus music selection influence emotional appraisal of INMI content? | Successful induction of INMI led to different subjective assessment: negative music led to more distressing and infrequent INMI than positive music. | 143, N |
| Moseley et al. 2018 | Phen | Survey | How do INMI compare with musical hallucinations? | Hallucinations are less frequent, less controllable, seldom lyrical, and less familiar than INMI tunes | 255, N |

Supplementary Table 2. Publications included after screening but excluded for ineligibility or being out of scope. Publication type indicates whether the study was published in a Journal (J), Conference proceedings (C), or Book/thesis format (B). Publications listed in order of publication, from the earliest to the most recent.

| **Citation** | **Pub. type** |
| --- | --- |
| Kellaris 2001 | C |
| Bennett 2003 | C |
| Kellaris 2003 | C |
| Bailes 2006 | J |
| Hemming 2008 | B |
| Liikkanen 2008 | C |
| Hemming 2009 | B |
| Liikkanen 2009 | C |
| Floridou, Williamson, and Müllensiefen 2012 | C |
| Liikkanen 2012b | J |
| Williamson and Müllensiefen 2012 | C |
| Liikkanen and Raaska 2013 | J |
| Bailes 2015 | J |
| Floridou 2015 | B |
| Hemming and Merrill 2015 | J |
| Williams 2015 | J |
| McNally-Gagnon 2016 | B |
| Lancashire 2017 | C |
| Liikkanen 2018 | B |
| Cotter and Silvia 2019 | J |
| Huovinen and Tuuri 2019 | J |
